# Passive Smoking Exposure and Perceived Health Status in Children Seeking Pediatric Care Services at a Vietnamese Tertiary Hospital

**DOI:** 10.3390/ijerph17041188

**Published:** 2020-02-13

**Authors:** Chau Quy Ngo, Giap Van Vu, Phuong Thu Phan, Hanh Thi Chu, Lan Phuong Thi Doan, Anh Tu Duong, Quan-Hoang Vuong, Manh-Tung Ho, Minh-Hoang Nguyen, Hong-Kong T. Nguyen, Hai Thanh Phan, Giang Hai Ha, Giang Thu Vu, Kiet Tuan Huy Pham, Tung Hoang Tran, Bach Xuan Tran, Carl A. Latkin, Cyrus S. H. Ho, Roger C. M. Ho

**Affiliations:** 1 Department of Internal Medicine, Hanoi Medical University, Hanoi 100000, Vietnam; ngoquychaubmh@gmail.com (C.Q.N.); vuvangiap@hmu.edu.vn (G.V.V.); thuphuongdr@gmail.com (P.T.P.); 2 Respiratory Center, Bach Mai Hospital, Hanoi 10000, Vietnam; chuthihanhbmh@gmail.com (H.T.C.); phuonglandoanthibm@yahoo.com.vn (L.P.T.D.); duongtuanh0802@gmail.com (A.T.D.); 3 Centre for Interdisciplinary Social Research, Phenikaa University, Yen Nghia, Ha Dong, Hanoi 100803, Vietnam; hoang.vuongquan@phenikaa-uni.edu.vn (Q.-H.V.); tung.homanh@phenikaa-uni.edu.vn (M.-T.H.); 4 Faculty of Economics and Finance, Phenikaa University, Yen Nghia, Ha Dong, Hanoi 100803, Vietnam; 5 Graduate School of Asia Pacific Studies, Ritsumeikan Asia Pacific University, Beppu, Oita 874-8577, Japanhtn2107@caa.columbia.edu (H.-K.T.N.); 6 Vuong & Associates Co., Hanoi 100000, Vietnam; 7 Institute for Global Health Innovations, Duy Tan University, Da Nang 550000, Vietnam; phanthanhhai9@duytan.edu.vn; 8 Faculty of Medicine, Duy Tan University, Da Nang 550000, Vietnam; 9 Faculty of Pharmacy, Duy Tan University, Da Nang 550000, Vietnam; 10 Center of Excellence in Evidence-based Medicine, Nguyen Tat Thanh University, Ho Chi Minh City 700000, Vietnam; giang.coentt@gmail.com; 11 Institute for Preventive Medicine and Public Health, Hanoi Medical University, Hanoi 100000, Vietnam; phamhuytuankiet_vkt@fpt.vn (K.T.H.P.); bach.ipmph@gmail.com (B.X.T.); 12 Institute of Orthopaedic and Trauma Surgery, Vietnam-Germany Hospital, Hanoi 100000, Vietnam; tranhoangtung.vd@gmail.com; 13 Bloomberg School of Public Health, Johns Hopkins University, Baltimore, MD 21205, USA; carl.latkin@jhu.edu; 14 Department of Psychological Medicine, National University Hospital, Singapore 119074, Singapore; cyrushosh@gmail.com; 15 Center of Excellence in Behavioral Medicine, Nguyen Tat Thanh University, Ho Chi Minh City 700000, Vietnam; pcmrhcm@nus.edu.sg; 16 Institute for Health Innovation and Technology (iHealthtech), National University of Singapore, Singapore 119077, Singapore; 17 Department of Psychological Medicine, Yong Loo Lin School of Medicine, National University of Singapore, Singapore 119228, Singapore

**Keywords:** secondhand smoking, health behaviors, children health, perceived health, Vietnam, passive smoking

## Abstract

Understanding the predictors of health conditions and exposure to secondhand smoke among children is necessary to determine the severity of the issues and identify effective solutions. Despite the significant prevalence in smoking and child exposure to secondhand smoke, there have been only a few studies focusing on this area in Vietnam, and thus the current study aims to fill in this gap. The questionnaires of 435 children aged between 0 and 6 and their caregivers, who agreed to participate in the research, were collected at the Pediatric Department of Bach Mai hospital, Hanoi, in 2016. Multivariable logistic regression was employed to identify factors associated with perceived health status and exposure to secondhand smoke among children in the last 24 h and the last 7 days from the date of the survey. Our study found that 43% of the respondents had smokers in the family, and 46.4% of children were exposed to passive smoking in the last 7 days. Urban children were most frequently exposed to passive smoking at home and in public, whereas in the rural area, the home and relatives’ houses were the most common places for exposure. Compared to children whose caregivers were farmers, children of non-government workers were more likely to be exposed to passive smoking in the last 7 days. Moreover, children in a family having smoking rules and no smokers were less likely to be exposed to passive smoking in the last 24 h and 7 days than those living in a family allowing smoking and having smokers. In conclusion, our study shows that the government needs to implement better public smoking monitoring and encourage caregivers to implement smoke-free households or smoking rules in their houses.

## 1. Introduction

Passive smoking exposure among children is widespread around the world and remains a considerable public health problem. It has been reported that 40–50% of children worldwide are regularly exposed to passive smoking, and children account for 28% of the 600,000 secondhand smoke-related deaths annually [1,2]. Exposure to environmental tobacco smoke is associated with numerous health risks in children, such as elevated blood pressure [3,4], dental decay [5], otitis media with effusion [6,7], pediatric asthma [8], childhood respiratory disease [9,10], pneumonia [11], and a heightened risk for sensorineural hearing loss [12]. Particularly, in-home passive exposure to smoke is found to increase the carotid intima-media thickness and arterial stiffness, which are among the major risk factors for cardiovascular disease [1,3,4,13]. In terms of childhood respiratory disease, besides increasing the risk of allergic rhinitis, when compared to non-exposed children, children with a history of passive exposure to smoke are also found to have defective interferon-γ production, which increases the susceptibility to the recurrence of respiratory infections [14,15]. Additional studies have pointed out that passive smoking increases exposure to airborne nicotine, tobacco’s main psychoactive substance, which could compound the illness of children hospitalized with influenza [16], asthma [1,17,18], or chronic kidney disease [19], and over a long period, could be a risk factor for smoking uptake in adolescents [20,21]. More importantly, passive exposure to smoke can lead to a higher risk of lung cancer, and people who were first exposed to passive smoking at a younger age are more likely to have lung cancer [22,23].

Despite the glaring problems caused by passive smoking for children, research on the factors associated with children’s exposure to environmental tobacco smoke appear to be largely focused on developed countries [24]. According to a systematic review on the predictors of children’s passive smoking exposure at home, Orton et al. [25] grouped the factors into five main categories: (1) socioeconomic status, which includes income, employment, and health insurance type; (2) parental characteristics (education, age, race/ethnicity); (3) family and home characteristics (family size, family structure, home environment); (4) child characteristics (age, sex); and (5) parental smoking characteristics (smoking behavior, attitudes, and efforts to quit smoking). The authors concluded that the strongest predictor is parental cigarette smoking status, and more notably, low socioeconomic status and being less educated were frequently and consistently linked with children’s passive smoking exposure at home. [25] Such findings have been echoed in other studies, which listed low parental education, unemployment and poverty [26,27], parental smoking behavior, dwelling space, and social and education status as risk factors [28,29].

Given the long list of confirmed health risks for children in terms of passive smoking and the subsequently high disease burden in adulthood [24], the World Health Organization (WHO) has launched a Framework Convention on Tobacco Control (FCTC) aimed at reducing tobacco consumption and passive smoking exposure at the national level [2]. A comprehensive review by Faber, Kumar, Mackenbach, Millett, Basu, Sheikh, and Been [24] has shown a gap in the literature on tobacco control effects in low- and middle-income countries, as well as a lack of research on child health focus in this area. 

The case of Vietnam is expected to resonate with other developing countries whose populations also struggle to protect children from environmental tobacco smoke and reduce the burden of smoke-related diseases [30]. In Vietnam, the WHO FCTC and the tobacco-free initiative MPOWER were implemented in March 2005 and 2008, respectively [31]. Since 2013, Vietnam has also issued and enforced a law that prohibits smoking in workplaces and public spaces, in addition to banning tobacco advertisements and requiring pictorial, graphic health warnings on cigarette packs [31]. However, according to official statistics, almost half of the children aged 13–15 in Vietnam are exposed to passive smoking at home [32], and there are 44,000 excess hospital admissions due to pneumonia each year among children aged under five years [11]. In terms of hair nicotine concentration, a study found an average of 1.21 ng/mg in children in Vietnam, which falls in the midrange for the 31 survey countries and indicates the closeness of interaction of the children with smoking household members [33]. Given the severity of the exposure to passive smoking among children, the current research strives to answer the following research questions:

- What is the difference regarding the characteristics of passive smoking exposure between urban and rural children?
- What are the associated factors of passive smoking exposure among children?

The results of this study are expected to provide insights into the current situation of passive smoking exposure among children in Vietnam and recommend preventive measures to reduce the exposure prevalence among children in Vietnam as well as other emerging countries that have a similar context.

## 2. Materials and Methods 

### 2.1. Study Designs

We performed a cross-sectional study from July to August 2016 with 435 children and caregivers at the Pediatric Department of Bach Mai Hospital, Hanoi, Vietnam. The Bach Mai hospital is the largest general hospital in Vietnam. A convenient sampling method was used to recruit children and their caregivers to the study. They were eligible to participate if they met the following inclusion criteria: (1) children were aged from 0–6 years old, (2) caregivers had normal cognition and able to answer the interview within 15–20 min, and (3) caregivers agreed to give their written informed consent. A total of 450 eligible children and their caregivers were approached, of which 435 children and caregivers agreed to participate (98.7%). Data of people refusing to enroll were not collected. 

### 2.2. Measurements

Data collection was performed within working hours (from 8:00 a.m. to 5:00 p.m. Monday–Friday) during the study period. Children and their caregivers were approached after their appointment by the data collectors who were medical students and nurses at the Bach Mai hospital. They were initially asked to identify the eligible criteria. After that, if they fulfilled the inclusion criteria, both children and caregivers were invited to a private room for an interview to assure their confidentiality and comfortability. They were introduced about the study purposes and their rights that they could withdraw from the study at any time without any influences on their current treatment and care. A structured questionnaire was built for face-to-face interviews with caregivers. This questionnaire was piloted in 10 caregivers and children admitted to the department and revised after receiving feedback from these participants regarding text, language, and logical order of questions. 

Primary outcomes: In this study, the primary outcome was passive smoking exposure. Caregivers were asked about whether their children were exposed to passive smoking in the last 24 h, and place where the children were exposed to passive smoking in the last 7 days. 

Secondary outcomes: We asked caregivers about whether they heard about passive smoking, their perceptions about effects of passive smoking on children’s health and diseases, their responses when seeing smokers around their children, and their perceived necessity of avoiding smoking cigarette before children. These items were adopted from the Global Youth Tobacco Use Survey in Vietnam [34].

Covariates: Caregivers were then interviewed to collect information of concerns including socio-demographic characteristics (age, education, occupation, living location), their relationship with the child and children’s information (age, sex), the number of smokers living in their family, the number of cigarettes used per week, smoking rules at home, and whether smoking was allowed in all rooms or not.

### 2.3. Statistical Analysis

Stata software version 14.0 was used to analyze the data. Chi-squared and Fisher’s exact tests were utilized to compare different characteristics between urban and rural. Mann–Whitney test was employed to measure the difference of continuous variables between two settings due to non-normal distribution. Multivariate logistic regression was employed to identify associated factors with passive smoking exposure among children in the last 24 h and the last 7 days. Potential independent factors included sociodemographic characteristics of children and caregivers (age and sex of children; age, sex, level of education, and occupation of caregivers; living location; the number of members in the family), ever heard about passive smoking, smoking rules at home, and having smokers in family. Stepwise forward selection strategy was applied to build the reduced regression models. Only variables with a *p*-value of the log-likelihood test less than 0.2 were selected and presented in the final models. Results of variance inflation factors (VIFs) test showed no collinearity among variables in the regression models (VIFs < 10). As for the multiplicity, Bonferroni adjustment was applied. In this study, our model had 11 hypothetic associated factors; thus, an adjusted *p*-value = 0.05/11–0.005 was used to detect statistical significance in the regression models. However, a *p*-value of less than 0.05 was also considered to imply potential difference and association. 

### 2.4. Ethical Approval

The approval of the Institutional Review Board was obtained through the Vietnam Respiratory Society (10/QD-VNRS).

## 3. Results

### 3.1. Sociodemographic Characteristics

Among 435 caregivers, the mean age was 34.1 (SD = 9.6) years old. The majority of them were from an urban area (70.8%), female (76.3%), and mothers of children (67.6%). Over half of the caregivers had university/college education or above (60.1%). The percentage of caregivers being officials in a non-governmental agency and having a small business were the highest with 29.2% and 23.5%, respectively. Differences between urban and rural were found in the sex of caregivers, level of education, occupation, number of family members, and age of the child (Table 1). Notably, because the study was conducted in a hospital setting, we also provide the prevalence of the health status of the participants (children) for further reference (Table A1). 

### 3.2. Passive Smoking Exposure

Regarding passive smoking exposure (Table 2), 43.0% reported having smokers in the family. The rate of caregivers reporting that smoking was not allowed at home was 65.7%, of which 22.5% had some exceptions for smoking at home. There were 8.5% of caregivers indicating that smoking was allowed in all rooms. In total, 19.3% of caregivers reported that their children were exposed passive smoking at home in the last 24 h. In addition, 46.4% of children were reported as being exposed to passive smoking in the last 7 days. 

Table 3 reveals that the majority of caregivers have heard about passive smoking, and 97.7% knew that passive smoking negatively affected children’s health. The most common diseases related to passive smoking that were reported were lung diseases (92.9%), lung cancer (79.5%), and other cancers (36.4%). Most caregivers stated that it was very necessary to avoid smoking before children (74.5%), and they would remind smokers to stop smoking and take children to other places (55.8%).

### 3.3. Associated Factors with Passive Smoking Exposure

Table 4 shows that children with caregivers who worked in a non-government agency were more likely to be exposed to passive smoking in the last 7 days (OR = 2.25; 95% CI = 1.02–4.99) compared to those working as farmers. Children with caregivers with high school education were more likely to be exposed to passive smoking in the last 24 h compared to those parents with less than high school education (OR = 2.35, 95% CI = 1.01–5.48). Never allowing smoking at home or not having smokers in the family may result in a lower likelihood of exposure to passive smoking in the last 24 h and in the last 7 days among children. Higher age of children increased the likelihood of exposure to passive smoking in the last 7 days (OR = 1.23; 95% CI = 1.07–1.41).

## 4. Discussion

This study is one of the first attempts to examine the prevalence and predictors of passive smoking exposure in children using pediatric care service in a Vietnamese hospital. Our findings indicate differences in the prevalence of passive smoking exposure between children from urban and rural areas. Moreover, some determinants of passive smoking exposure were also found, such as caregiver’s occupation and smoking rules in the family.

Our findings show a high prevalence of exposure to passive smoking among children visiting the hospital (46.4%) and a difference in the location in which urban and rural children are usually exposed to passive smoking. In particular, the home and public places are locations that urban children are most frequently exposed to passive smoking (18.5% and 18.2%, respectively), whereas the home and relatives’ houses are places that rural children are most frequently exposed to passive smoking (34.7% and 11%, respectively). Compared to 53.5% of the prevalence of passive smoking exposure among adult non-smokers at home in Vietnam reported by The Global Adult Tobacco Survey (GATS) [2], the prevalence of children exposed to passive smoking in this study is significantly lower at 23.2%. Nevertheless, this comparison should only be seen as a point of reference, because the prevalence reported by GATS was during the last 30 days, whereas the prevalence in our study was during the last 7 days. 

These results can be explained by the difference between urban and rural families in having smokers in family and smoking rules. Specifically, our findings also reveal that the percentage of families having at least one smoker is relatively high, at 38.3% in urban families and 54.3% in rural families, and that smoking is more loosely controlled in rural families than in urban families. The proportions of urban families prohibiting smoking at home and allowing smoking in all rooms were 48.4% and 5.66%, respectively, whereas those proportions of rural families were 30.7% and 13.64%. This difference might be due to differences in educational level and general knowledge between urban and rural residents. Rural residents tend to have a lower educational level than their urban counterparts, and fewer rural caregivers have accurate knowledge and perceptions regarding of effects of passive smoking on children [25]. 

The result of this study confirms findings in other studies that rural children are more likely to be exposed to passive smoking at home than children living in an urban area [35,36]. For children in an urban area, besides home, a public place is also a common place for passive smoking exposure. This stands in sharp contrast to the fact the Vietnamese government has banned public smoking [37], which suggests that the government needs to put more efforts into curbing public smoking. Changing behaviors of millions of people is not easy; thus, to be more effective in curbing public smoking in Vietnam, more attention should be paid to evidence-based policies such as the application of behavioral economics in public intervention policies [38,39,40]. 

There are several predictors of exposure to passive smoking among children that can be drawn from this study, namely, caregiver’s occupation and education, family smoking rule, having a smoker in the family, and child’s age. Children whose caregivers worked in non-governmental sectors were more likely to be exposed to passive smoking than those whose caregivers were farmers. The result was contrasted to the previous finding in a national survey on secondhand smoke exposure among Vietnamese youths, which showed that children having parents as farmers were more likely to be exposed to SHS than parents having other jobs [41]. This might be due to the small sample size in our study and the difference in the age of the studied children. Moreover, our sample was recruited from a hospital setting, whereas this survey was performed in a community setting. 

In terms of family characteristics, children in a family not allowing smoking at home were reported to have less chance of exposure to passive smoking than those in a family allowing smoking. Similarly, families having smokers increased the chance of exposure to passive smoking among children in the household. The findings are consistent with the result of a study on the determinants of passive smoking exposure among pregnant women in an urban setting in Vietnam [3]. These two findings, in turn, confirm the importance of family’s characteristics in reducing exposure to passive smoking among children [42] and suggest that policymakers examine methods to improve the effectiveness of programs aimed at raising the awareness of negative impacts of passive smoking in families having children [43]. Moreover, we also found that along with the high prevalence of caregivers not acquiring knowledge of passive smoking (approximately 40%), urban caregivers had more knowledge regarding passive smoking and considerate responses when the children were exposed to smoke than the rural caregivers. Such a high prevalence might lead to higher passive smoking prevalence among children due to the lack of awareness and preparedness. Thus, the result underlines the necessity to promote the public awareness and perform educational interventions about passive smoking among caregivers and children, especially those in the rural area. 

Age of children is also found to be a significant predictor of exposure to passive smoking; older children were found to have a higher chance of expose to passive smoking. This finding contributes evidence to the association between age and exposure to passive smoking among children aged 0 to 6 years. According to the systematic review of predictors of children’s passive exposure to smoke [25], three studies have similar results with our study, but their targets were mostly adolescents and infants less than 1-year-old. In the age range of 0 to 6 years, Vietnamese younger children, probably infants, are more likely to be kept away from public and crowded places for the purpose of safety. However, when they grow up, that tendency might decrease, and thus older children might be more susceptible to passive smoking exposure. Once again, the potential of more effective public smoking monitoring to reduce the risk for children is highlighted. 

This study is not without limitations. First, the convenience sampling method may be an obstacle in the generalization of the results. Because the study was conducted in a hospital setting, the results may be biased due to hospital visitors possibly being less healthy than the normal population (see Table A1). As a result, all the prevalence in the current study should only be viewed as a reference, but not for a generalizing purpose. Nonetheless, the associations investigated from the current study are less affected by the biases mentioned above, and thus generalization is possible. Nonetheless, future studies should aim at random sampling to confirm the prevalence and empirical associations. Second, due to the self-reported nature of our study, recall bias might arise when the subjects answer the survey. Finally, as this study employs the frequentist approach of statistical analysis, which has recently raised cautions among scientists worldwide [44], future studies should not merely employ frequentist approach, but also address this concern by applying Bayesian statistics for better validity and confirmation [45]. Given the limitations, the study has still provided a useful reference point for further research in this area in Vietnam.

## 5. Conclusions

In conclusion, our study has found that the implementation of a free-smoke household and no-smoking rule in a family can help reduce the exposure to passive smoking among children. The prevalence of caregivers not obtaining knowledge regarding passive smoking is relatively high, especially in rural areas. The home is the place where urban children are most frequently exposed to passive smoking, followed by public places, and thus the government needs to implement more effective measures to prohibit smoking in public places and non-governmental workplaces, as well as promote awareness about the negative effects of smoking and passive smoking in the countryside. More importantly, as the home was found to be the most common location of passive smoking exposure, the ways in which to encourage caregivers to implement smoke-free households or establish and enforce smoking rules in the home is an area that merits more attention from public health policymakers in Vietnam as well as scientists in other emerging countries where the context is similar. 

## Figures and Tables

**Table 1:** Sociodemographic characteristics of caregivers.

Characteristics	Urban		Rural		Total		*p*-Value
	*n*	%	*n*	%	*n*	%	
Total	308	70.8	127	29.2	435	100.0	
Gender of caregivers							
Male	62	20.1	41	32.3	103	23.7	0.01
Female	246	79.9	86	67.7	332	76.3	
Relationship with child							
Father	58	18.8	37	29.1	95	21.8	0.08
Mother	217	70.5	77	60.6	294	67.6	
Grandmother/grandfather	32	10.4	12	9.5	44	10.1	
Sister	1	0.3	1	0.8	2	0.5	
Level of education							
Primary school	3	1	6	4.7	9	2.1	<0.01
Junior high school	25	8.1	42	33.1	67	15.4	
High school	60	19.5	38	29.9	98	22.5	
University, college	196	63.6	40	31.5	236	54.3	
Postgraduate	24	7.8	1	0.8	25	5.8	
Occupation							
Farmer	9	2.9	39	30.7	48	11.0	<0.01
Official in state agency	70	22.7	14	11	84	19.3	
Official in non-governmental agency	100	32.5	27	21.3	127	29.2	
Small business, handmade jobs	74	24.0	28	22.1	102	23.5	
Others	55	17.8	19	15.0	75	17.0	
	Mean	SD	Mean	SD	Mean	SD	
Age of caregiver (years)	34.4	9.6	33.6	9.6	34.1	9.6	0.29
Number of members in family	4.5	1.4	4.8	1.5	4.6	1.4	0.04
Number of children from 0–6 years old in family	1.5	0.62	1.5	0.77	1.5	0.67	0.91
Age of child	2.79	1.47	3.24	1.78	2.92	1.58	0.02

**Table 2:** Passive smoking exposure among children attending pediatric care services.

Characteristics	Urban		Rural		Total		*p*-Value
	*n*	%	*n*	%	*n*	%	
Having smokers in family	118	38.3	69	54.3	187	43.0	0.002
Number of smokers in the family							
0	190	61.7	58	45.7	248	57.0	0.008
1	107	34.7	61	48.0	168	38.6	
2	11	3.6	8	6.3	19	4.4	
Smoking rules at home							
Allow smoking	18	5.8	14	11.0	32	7.4	0.005
Do not allow smoking, but with some exceptions	66	21.4	32	25.2	98	22.5	
Never	149	48.4	39	30.7	188	43.2	
No rules	75	24.4	42	33.1	117	26.9	
Smoking is allowed in all rooms	9	5.66	12	13.64	21	8.5	0.031
Child exposed to passive smoking in the last 24 h	55	17.9	29	22.8	84	19.3	0.214
Place where child exposed to passive smoking in the last 7 days							
Home	57	18.5	44	34.7	101	23.2	0.000
Relative’s/ friends’ house	17	5.5	14	11	31	7.1	0.042
Car/motorbike	5	1.6	1	0.8	6	1.4	0.676
Public	56	18.2	5	3.9	61	14.0	0.000
Other	0	0	3	2.4	3	0.7	0.024
Not exposed	173	56.2	60	47.2	233	53.6	0.090
	Median	IQR	Median	IQR	Median	IQR	*p*-value
Number of cigarettes per week	35	3–90	42	20–105	35	10–100	0.097

**Table 3:** Knowledge and attitude of caregivers on passive smoking exposure among children attending pediatric care services.

Characteristics	Urban		Rural		Total		*p*-Value
	*n*	%	*n*	%	*n*	%	
Ever heard about passive smoking	212	68.8	64	50.4	276	63.5	<0.01
Passive smoking affects children’s health	302	98.1	123	96.9	425	97.7	0.49
Passive smoking-related diseases							
Cardiovascular diseases	109	35.5	39	30.7	148	34.1	0.34
Lung diseases	289	94.1	114	89.8	403	92.9	0.11
Lung cancer	251	81.8	94	74	345	79.5	0.07
Other cancers	119	38.8	39	30.7	158	36.4	0.11
Other	18	5.9	4	3.2	22	5.1	0.34
Necessity of avoiding smoke before children							
Very necessary	241	78.3	83	65.4	324	74.5	0.01
Necessary	66	21.4	42	33.1	108	24.8	
Unnecessary	1	0.3	2	1.6	3	0.7	
Responses when seeing smokers if children play around							
Remind smokers to stop smoking	30	9.8	12	9.5	42	9.7	0.04
Not remind smokers to stop smoking, take children to other places	99	32.3	39	30.7	138	31.8	
Remind smokers to stop smoking, take children to other places	174	56.7	68	53.5	242	55.8	
Do nothing	4	1.3	8	6.3	12	2.8	

**Table 4:** Associated factors with passive smoking exposure in the last 24 h and last 7 days among children.

Characteristics	Exposure to Passive Smoking in the Last 24 h				Exposure to Passive Smoking in the Last 7 Days			
	Odds ratio (OR)	*p*-Value	95% CI		Odds Ratio (OR)	*p*-Value	95% CI	
Gender of caregiver								
Male					REF			
Female					0.65	0.095	0.39	1.08
Age of caregiver (years)					0.98	0.105	0.96	1.00
Age of child (years)					1.23	0.004	1.07	1.41
Level of education of caregivers								
Secondary school or below	REF							
High school	2.35	0.048	1.01	5.48				
Above high school	1.53	0.328	0.65	3.59				
Occupation of caregivers								
Farmer	REF				REF			
Official in government agency	0.50	0.297	0.14	1.84	0.97	0.945	0.41	2.28
Official in non-government agency	1.43	0.510	0.49	4.17	2.25	0.045	1.02	4.99
Small business, handmade jobs	2.37	0.095	0.86	6.51	2.15	0.061	0.96	4.79
Others	1.36	0.576	0.47	3.95	1.90	0.128	0.83	4.33
Ever heard about passive smoking								
No					REF			
Yes					0.68	0.093	0.43	1.07
Smoking rules at home								
Allow smoking	REF				REF			
Do not allow smoking, but with some exceptions	0.56	0.213	0.22	1.40	1.21	0.687	0.48	3.04
Never	0.14	0.000	0.05	0.37	0.25	0.002	0.10	0.59
No rules	0.25	0.005	0.09	0.66	0.36	0.028	0.15	0.90
Having smokers in family								
Yes	REF				REF			
No	0.29	0.000	0.16	0.50	0.40	0.000	0.26	0.62

## Appendix A

**Table A1:** Health status of participants prior to the survey.

Characteristics	Urban		Rural		Total		*p*-Value
	*n*	%	*n*	%	*n*	%	
Ever being diagnosed any diseases	57	18.5	26	20.5	83	19.1	0.64
Having acute symptoms in the last 4 weeks							
Fever	191	62	56	44.1	247	56.8	<0.01
Cough	208	67.5	64	50.4	272	62.5	<0.01
Dyspnea	66	21.4	15	11.8	81	18.6	0.02
Expectoration	137	44.5	36	28.4	173	39.8	<0.01
Wheeze	94	30.5	30	23.6	124	28.5	0.15
Sniffle, rhinitis	170	55.2	49	38.6	219	50.3	<0.01
Red eye, allergic eyes	21	6.8	7	5.5	28	6.4	0.61
Otitis	17	5.5	5	3.9	22	5.1	0.49
Other	79	25.7	47	37	126	29.0	0.02
Having other health issues in the last 12 months	69	22.4	30	23.6	99	22.8	0.78
Perceived health status in the last 4 weeks							
Good	41	13.3	24	18.9	65	14.9	0.22
Medium	176	57.1	73	57.5	249	57.2	
Bad	91	29.6	30	23.6	121	27.8	
	Mean	SD	Mean	SD	Mean	SD	
Times of visiting health facilities in the last 12 months	3.8	4.1	2.9	3.6	3.5	4.0	0.02
